# Neonatal mortality and associated factors among neonates admitted to neonatal intensive care units at public hospitals in Addis Ababa, Ethiopia:

**DOI:** 10.1186/s12887-025-06218-y

**Published:** 2025-10-21

**Authors:** Getinet Tilahun Simeneh, Getaye Worku Tesema, Dawit Tarko Alamenie, Befikad Assefa Seifu, Merima Mohammed Hassen, Tigist Shiferaw Mekuriaw, Soliyana Hailu Chekol, Biniam Yohannes Wotango

**Affiliations:** 1 Health Service Quality and Patient Safety Directorate, Gandhi Memorial Hospital, Addis Ababa, Ethiopia; 2 National Data Management & Analytic Center, Ethiopian Public Health Institute, Addis Ababa, Ethiopia; 3 Training and Research Directorate, Gandhi Memorial Hospital, Addis Ababa, Ethiopia; 4 Department of Nursing and Midwifery, Saint Peter’s Hospital, Addis Ababa, Ethiopia; 5 Department of Nursing and Midwifery, Gandhi Memorial Hospital, Addis Ababa, Ethiopia; 6 Outpatient Department Directorate, Gandhi Memorial Hospital, Addis Ababa, Ethiopia

**Keywords:** Neonate, Neonatal mortality, Intensive care unit, Associated factors, Ethiopia

## Abstract

**Background:**

Globally, approximately 2.4 million neonatal mortalities occur each year, accounting for nearly half of all under-five mortalities. The neonatal period is the most vulnerable time for survival, in which newborns face the highest risk of death, and factors for neonatal mortality vary from place to place and time to time. This study aimed to assess neonatal mortality and its associated factors among neonates admitted to neonatal intensive care units at public hospitals in Addis Ababa, Ethiopia.

**Methods:**

An institution-based retrospective cross-sectional chart review was carried out at public hospitals in Addis Ababa, Ethiopia. The systematic sampling method was used to include 313 neonates who were admitted to the intensive care unit from 1st August 2023 to 30 July 2024. Data were collected via a structured checklist, entered into epi data 3.1, and then analyzed via the Statistical Package for Social Science (SPSS) version 25. Descriptive statistics were used to summarize the data. Bivariable and multivariable logistic regression analyses were employed with statistical significance declared at a p value < 0.05.

**Results:**

Twenty-seven (8.6%) neonates (95% CI: 5.8, 11.4) died. Mothers experienced hemorrhage (AOR = 2.89; 95% CI: (1.11, 7.36), pregnancy induced hypertension (AOR = 1.98; 95% CI: (1.23, 3.29), prolonged membrane rupture (AOR = 3.56; 95% CI: (1.19, 10.69), instrumental vaginal delivery (AOR = 5.74; 95% CI: (1.52, 12.78) and resuscitation (AOR = 3.69; 95% CI: (1.16, 11.75)) were significantly associated with neonatal mortality.

**Conclusion:**

The present study finding revealed a slightly lower neonatal mortality rates compared to the previous report findings in Ethiopia. However, neonatal mortality remains a critical public health concern, and thus, stakeholders need to develop interventions that all risk factors into consideration.

## Introduction

The World Health Organization (WHO) defines neonate and neonatal mortality (NM) as the period up to the first 28 days after live birth and death that occurs within 28 completed days of birth, respectively [1–3]. Globally, approximately 140 million births occur each year, with an estimated 2.6 million deaths within a month of age and approximately half (47%) of all deaths in children under the age of five [3, 4].

Reducing NM is a worldwide concern, as stated in the Millennium Development Goals (MDGs) and the Sustainable Development Goals (SDGs) (reduce NM to at least 12 per 1000 live births by 2030) [5]. However, the incidence of NM is unacceptably high; 2 million mortalities occurred globally in 2015, and more than 30 million neonatal deaths are expected to occur between 2017 and 2030. In addition, almost half of the mortalities are expected in sub-Saharan Africa [6]. Ethiopia is among the countries with the highest neonatal mortality rate (NMR), and according to the 2016 Ethiopian Demographic Health Survey (EDHS), it was estimated to be 29 per 10,000 live births [7] and 30 per 10,000 live births in 2019 [8].

Studies conducted in five countries (India, Pakistan, Nigeria, the Democratic Republic of the Congo and Ethiopia alone) account for half of all neonatal deaths at the country level, i.e., 24%, 10%, 9%, 4%, and 3%, respectively [4, 9]. Studies in Africa have shown that the incidence of NMs among NICU-admitted neonates is high, accounting for 26.5%, 20.2%, 15.5% and 6.6% in Somalia, Ghana, Egypt and Eritrea, respectively [10–13], and studies revealed that in Ethiopian public health facilities; Mizan Tepi University Teaching Hospital, Debre Markos Hospital, and St. Luke Wolisso Hospital accounted for 22.8%, 21.3%, and 17% of NMs, respectively [14–16].

Despite global progress in reducing NM and actions such as developing global and national neonatal and child survival strategies and newborn health as a priority, efforts to improve progress are still needed to achieve the 2030 SDG target, especially in low- and middle-income countries (LMICs), including Ethiopia [13, 17]. Nearly all new-born deaths (99%) occur within the first 28 days of life, predominantly in the world’s poorest regions particularly in sub-Saharan Africa (SSA) and South Asia, where complications related to neonatal conditions and medical diagnoses account for the majority of neonatal mortality [18–20]. The Mini EDHS 2019 showed an increasing trend in neonatal mortality in Ethiopia, indicating ongoing challenges in newborn care and inadequate implementation of essential interventions [8]. The poor progress of neonatal and child wellness during facility-based care has led to slow progress in the attainment of improved health care delivery systems for the country [21, 22]. Mortalities among NICU-admitted neonates have been linked to several predictors, including mothers with complications of pregnancy, respiratory distress syndrome, neonatal sepsis, gestational ages less than 28 weeks, low APGAR scores, home deliveries, jaundice, hypoglycemia, and very low birth weight, which are associated with not only a burden on the image of the country but also devastating social, psychological, and economic factors related to the quality of the family and community life [19, 20, 23].

Approximately half of these preventable deaths could be avoided by providing quality antenatal care, skilled birth attendance for all births, and specialized care for small and sick newborns. Unfortunately, studies assessing neonatal mortality among NICU-admitted neonates in the study area are limited. Thus, determining and identifying factors for neonatal mortality is crucial for prioritizing the development of targeted and evidence-based health interventions.

## Methods

### Study design and setting

An institution-based retrospective cross-sectional chart review was conducted among neonates admitted to the neonatal ICU at four public hospitals in Addis Ababa, Ethiopia, from 1 st August 2023 to 30 July 2024. Addis Ababa has twelve public hospitals, and eleven of these hospitals have neonatal intensive care units. Neonatal health services are provided in both public and private healthcare care facilities, and approximately 200,000 births occur each year in Addis Ababa [8]. Therefore, the study was conducted in four randomly selected public hospitals of Addis Ababa, i.e., Abebech Gobena Maternal and Children Hospital (AGMCH) (an affiliate of Yekatit 12 Hospital Medical College), Gandhi Memorial Hospital (GMH), Menelik II Comprehensive Specialized Hospital (MCSH) and Tikur Anbesa Specialized Hospital (TASH). All four hospitals provide neonatal intensive care services, and the number of admitted neonates varies from time to time and from hospital to hospital, with an annual average of 7560 neonatal admissions. The NICUs in the four hospitals are also staffed with pediatricians, neonatologists (only in TASH), Resident Doctors, Intern Doctors and Nurses.

### Study population and sample size

All neonates who were admitted to the NICU in four public hospitals in Addis Ababa, Ethiopia, from 1 st August 2023–30 July 2024.

The minimum sample size required for this study was calculated using both specific objectives, i.e., the single population proportion formula and the associated factors. Then the largest sample size was obtained from the first objective by a single population proportion formula considering the assumption that the proportion of neonatal mortality from the previous study was 13.9% [4]. The confidence level is 95%, and the margin of error is 4% (d = 0.04), calculated as follows.

$$\mathrm{Sample}\;\mathrm{size}\;\mathrm n=\frac{\left(\mathrm Z{\displaystyle\frac{\mathrm\alpha}2}\right)^2\ast\mathrm P\left(1-\mathrm P\right)}{\mathrm d^2}$$

$$\frac{{(1.96)}^{2\ast}(0.14\ast(1-0.14))}{\left(0.04\right)^2}=289$$

Then to allow for incomplete neonatal charts we added 10%, given final required sample ≈ 318.

Therefore, the large sample size was 289 plus 10% for the possible incomplete charts (29), i.e., a total of 318 samples were taken.

### Sampling procedure

 There are twelve public hospitals in Addis Ababa. With the exception of Amanuel Mental Hospital, all other hospitals provide neonatal ICU services. From the eleven hospitals giving neonatal ICU care, four were randomly selected for this study. To include 318 neonates, a proportional allocation method was made based on the annual average of 7,560 neonatal admissions in the four hospitals, ensuring a representative sample while maintaining feasibility for data collection.

Then, a systematic random sampling technique was used for the enrollment of 318 neonates under the following assumptions: N (the estimated annual number of admitted neonates was 7560) and n (the minimum sample size needed, including a 10% nonresponse rate = 318), which gives a sampling fraction (k): where k = N/n = > 7560/318 ≈ 24. Accordingly, medical records of the neonates were listed, and every 24th record was chosen for the sample. The first 24 records from each hospital were numbered 1 through 24 so that data collectors could figure out where to begin. Then, one number was randomly selected from each hospital using lottery method as the starting point. For instance, if the number 4 was drawn, data collection would begin from the 4th record and then proceed by selecting every 24th record until the required sample size of 318 was attained across all hospitals.

### Operational definition

#### Neonatal mortality (NM) 

The death of live newborns within 28 days of age [4, 24].

##### Hypothermia

Neonates with a low body temperature (< 36.5 °C) who were diagnosed and recorded on neonatal charts during the admission period [25].

##### Prematurity

Living neonates delivered before 37 completed weeks of gestation who were diagnosed and recorded on neonatal charts during the admission period [25, 26].

##### Birth asphyxia

Neonate diagnosed with an Apgar score of < 6 in the fifth minute, does not cry immediately after birth, and experiencing respiratory distress, floppiness, loss of consciousness, and neonatal reflexes [26].

### Data collection procedures

Data were collected from newborns’ medical records via a checklist adapted from the WHO document of review and audit of neonatal death and other related literature with some modifications on the basis of research objectives [18, 20, 23, 24, 26]. The checklist was prepared in English. Eight well-trained nurses and two experienced health officers were recruited as data collectors and supervisors, respectively.

### Data quality assurance

The quality of the tool was assured by training data collectors and supervisors and pretesting the tool with 5% of the sample size at Zewditu Memorial Hospital (one of the twelfth hospitals in Addis Ababa), which is not included in the study area. All the questionnaires were checked for completeness. During the data collection period, the data collectors were supervised. There were meetings between the data collectors and the principal investigator in which problematic issues arising during data collection and any errors encountered were discussed. The collected data were reviewed again and checked for completeness before entry.

### Data entry and analysis

After checking for data completeness, the data were cleaned, coded, entered into Epi data 3.1 and exported to the SPSS version 25 software package for analysis. Descriptive statistics were computed to summarize maternal, obstetric and neonatal, characteristics. Continuous variables were categorized and categorical variables were included based on completeness of data and results were presented by frequency and percentages. The assumptions of logistic regression were checked before analysis. Bivariable logistic regression was performed to assess the crude relationship between the independent variables and the dependent variable at *P* < 0.25. Multivariable logistic regression was employed to determine the independent effect of each variable on the outcome variable. The fitness of the model was subsequently checked using the Hosmer and Lemeshow test at *P* > 0.5. The results are presented in the form of texts, tables, and figures. The degrees of association between variables were determined by odds ratios and significance levels at 95% confidence intervals.

### Ethical consideration

Ethical approval was obtained from Addis Ababa Medical and Business College, the School of Public Health Institution Review Board (IRB). The study was also granted permission by the Addis Ababa City Administration Health Bureau (AACAHB). As this was a retrospective records review, the need for informed consent was waived by the IRB and permission to access neonatal records was obtained from NICU department.

## Results

### Maternal socio demographic characteristics

A total of 318 ICU-admitted neonatal records were identified for review, and 313 records were reviewed, for a review rate of 98.4%. From the reviewed charts, 159 (50.8%) were in the age group of 25–34 years, with a mean age of 27.63 years (± SD 4.981) and minimum and maximum ages of 19 and 41, respectively. Approximately two-thirds of the study participants, 205 (66.5%), were permanently living in Addis Ababa, and 285 (91.1%) women were married. With respect to the educational status of the maternal characteristics, nearly half 150 (48.0%) had attended secondary school and above, and 17 (5.4%) of others’ educational statuses were not applicable (Table 1).

**Table 1:** Maternal socio demographic characteristics of ICU -admitted neonates in addis Ababa, Ethiopia, 2024 (n = 313) [27]

Background characteristics	Category	Frequency (*n*)	Percent (%)
Age of the mother	15–24 years	95	30.4
	25–34 years	159	50.8
	>=35 years	59	18.8
Residence	Addis Ababa	205	65.5
	Out of Addis Ababa	108	34.5
Marital status of the mother	Single	12	3.8
	Married	285	91.1
	Others *	16	5.1
Educational status of the mother	No formal education	48	15.3
	Primary education	98	31.3
	Secondary and above	150	48.0
	Not applicable	17	5.4

- Others * =divorced, widowed, and not applicable.

### Maternal medical and obstetric-related characteristics

Among the total reviewed charts, 299 (95.5%) mothers had ANC contact during their recent pregnancy; among them, more than two-thirds 212 (67.7%) had four or more ANC contacts, and 308 (98.4%) mothers were tested for VDRL, with 97.1% having nonreactive results. The majority 288 (92.0%) of the participants had singleton pregnancies, and more than half of them 170 (54.3%) had given birth via SVD, with more than three-fourths 238 (76.0%) of them had given birth at hospitals. Half of the maternal characteristics 161 (51.4%) were multiparous, and more than three-fourths 130 (77.4%) had two or more years of birth interval. One hundred thirty-eight (44.1%) mothers experienced pregnancy-related complications, and the most common complication during pregnancy was pregnancy-induced hypertension 81 (58.7%)), followed by hemorrhage 38 (27.5%)) (Table 2).

**Table 2:** Maternal medical and obstetric-related characteristics of ICU-admitted neonates in addis Ababa, Ethiopia: (*n* = 313)

Background characteristics	Category	Frequency (*n*)	Percent (%)
ANC contact	No ANC contact	14	4.5
	< 4	87	27.8
	≥ 4	212	67.7
VDRL	Reactive	4	1.3
	Nonreactive	304	97.1
	NA	5	1.6
Birth type	Single	288	92.0
	Multiple	25	8.0
Mode of delivery	SVD	170	54.3
	IVD	25	8.0
	C/D	118	37.7
Delivery place	Health center	64	20.5
	Hospital	238	76.0
	Others^**^	11	3.5
Parity	Primipara	145	46.3
	Multipara	161	51.4
	Grand multi Para	7	2.3
Birth interval (*n* = 168)	< 2 years	110	16.1
	≥ 2 years	130	77.4
	NA	11	6.5
PROM	Yes	81	25.9
	No	232	74.1
Pregnancy-related complications	Yes	138	44.1
	No	175	55.9
Pregnancy-related complications (*n* = 138)	Hemorrhage	38	27.5
	PIH	81	58.7
	Others ^***^	19	13.8
Chronic diseases	Yes	17	5.4
	No	296	94.6
Types of chronic diseases (*n* = 17)	Chronic hypertension	8	47.1
	Diabetes mellitus	4	23.5
	Others****	5	29.4

*ANC *antenatal care, *NA *not applicable, *SVD *spontaneous vaginal delivery, *IVD *instrumental vaginal delivery, *C/D *cesarean delivery, others^**^_=_ home and private, PIH_=_pregnancy-induced hypertension, others ***_=_ gestational diabetes mellitus, oligohydramnios and Rh incompatibility, others****= HIV, cardiac diseases

### Neonatal-related characteristics

Among all the neonates studied, more than half 179 (57.2%) had normal birth weights and regarding gestational age; five out of seven 227 (72.5%) neonates were term. More than four in five 258 (82.4%) neonates were admitted within a day after delivery, and the majority 249 (79.6%) stayed in the hospital for less than seven days. With respect to the APGAR scores of the neonates, more than half (55.0% and 63.9%) had APGAR scores between 7 and 10 at the 1 st and 5th minutes, respectively (Table 3).

**Table 3:** Neonatal characteristics of ICU-admitted neonates in addis Ababa, Ethiopia, 2024: (*n* = 313**)**

Background characteristics	Category	Frequency (*n*)	Percent (%)
Baby sex	Male	152	48.6
	Female	161	51.4
Baby weight at admission	LBW	125	39.9
	Normal weight	179	57.2
	Macrosomic	9	2.9
Baby age at admission	<= 1 day	258	82.4
	> 1 day	55	17.6
Baby GA	Preterm	75	24
	Term	227	72.5
	Post term	11	3.5
APGAR score within 1 st minutes	Low (< = 3)	49	15.7
	Moderate (4–6)	92	29.3
	Normal (> = 7)	172	55.0
APGAR score within 5th minutes	Low (< = 3)	15	4.8
	Moderate (4–6)	98	31.3
	Normal (> = 7)	200	63.9
Resuscitation given	Yes	110	35.1
	No	203	64.9
Time of initiation of EBF	Within 1 hour	130	41.5
	After 1 hour	164	52.4
	not at all	19	6.1
Length of stay at the facility	< 7 days	249	79.6
	>=7days	64	20.4

*APGAR *appearance, pulse, grimace, activity, and respiration, *EBF *exclusive breastfeeding

### Magnitude of NM

**Fig. 1:**
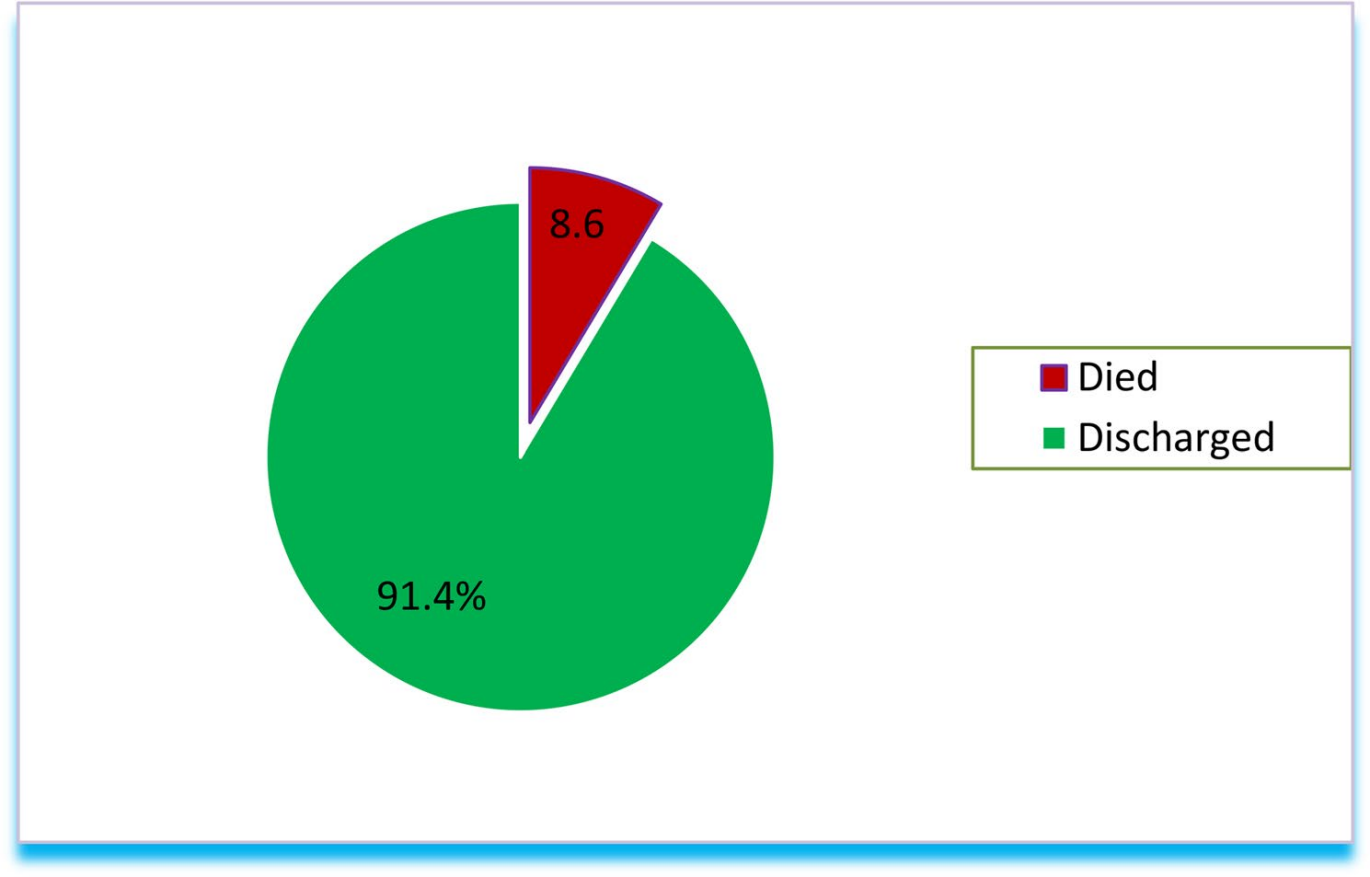
- Magnitude of neonatal mortality among ICU-admitted neonates in Addis Ababa, Ethiopia, 2024: (*n* = 313)

Among the neonates included in this study, 27 (8.6%) (95% CI: 5.8, 11.4) died during the study period (Fig. 1).

### Factors associated with neonatal mortality among NICU-admitted neonates

In the bivariable analysis, ANC contacts of the mother, complications during pregnancy (hemorrhage and PIH), instrumental vaginal delivery, sex of the baby, PROM, APGAR score within 5 min of the baby, a baby who underwent resuscitation and EBF were associated with NM at a p value of less than 0.25. A total of 8 explanatory variables with P values < 0.25 in the bivariable logistic regression analysis were subsequently regressed against NM. Thus, in the multivariable logistic regression analysis, complications during pregnancy, PROM, instrumental vaginal delivery, and being resuscitated were significantly associated with NM at a P value of < 0.05.

Accordingly, newborns whose mothers experienced hemorrhage and PIH during pregnancy were 3 times and 2 times more likely, respectively, to experience neonatal mortality than those whose mothers had no pregnancy complications [AOR = 2.89; 95% CI: (1.11, 7.36); *p* = 0.03], and [AOR = 1.98; 95% CI: (1.23, 3.29); *p* = 0.02], whereas the odds of neonatal mortality were 3.56 times [AOR = 3.56; 95% CI: (1.19, 10.69), *p* = 0.02] greater among neonates born to mothers with PROM than among their counterparts.

Compared with neonates who delivered through spontaneous vaginal delivery, those who delivered through instrumental vaginal delivery were more than 5-folds more likely to die [AOR = 5.74; 95% CI: (1.52, 12.78); *p* = 0.01]. The odds of death among neonates who were resuscitated were more than 3 times greater than those among neonates who were not received resuscitation [AOR = 3.69; 95% CI: (1.16, 11.75), *p* = 0.02] (Table 4).

**Table 4:** Factors associated with NM among ICU-admitted neonates in addis Ababa, Ethiopia, 2024: (*n* = 313)

Variables		NM		COR with 95% CI	AOR with 95% CI
		Yes N (%)	No N (%)		
ANC contact (s)	No ANC contact	2 (14.3)	12 (85.7)	3.37 (0.66, 17.12)	4.91 (0.72,13.27)
	< 4	15 (17.2)	72 (82.8)	4.208 (1.81, 9.79)	2.55 (0.93, 7.05
	>= 4	10 (4.7)	202 (95.3)	Ref.	Ref.
5th minute APGAR score	Low	2 (13.3)	13 (86.7)	2.41 (1.0, 15.48)	1.25 (0.21, 4.42)
	Moderate	13 (13.3)	85 (86.7)	2.40 (1.20, 6.75)	1.44 (0.40, 5.19)
	Normal	12 (6.0)	188 (94.0)	Ref.	Ref.
Sex of the baby	Male	18 (11.8)	134 (88.2)	2.27 (0.99, 5.22)	1.68 (0.62, 4.56)
	Female	9 (5.6)	152 (94.4)	Ref.	Ref.
Mode of delivery	SVD	9 (5.3)	161 (94.7)	Ref.	Ref.
	IVD	7 (28.0)	18 (72.0)	6.96 (2.31, 20.93)	5.74 (1.52, 12.78)**
	C/S	11 (9.3)	107 (90.7)	1.84 (0.74, 4.59)	1.64 (0.58, 4.66)
Complication during pregnancy	Hemorrhage	5 (13.2)	33 (86.8)	2.50 (1.67, 4.93)	2.89 (1.11, 7.36)*
	PIH	10 (12.3)	71 (87.7)	2.32 (1.23, 3.64)	1.98 (1.23, 3.29)*
	Other complications	2 (10.5)	17 (89.5)	1.94 (0.87, 4.62)	1.73 (0.67, 4.69)
	No complication	10 (4.0)	165 (94.0)	Ref.	Ref.
PROM	Yes	14 (17.3)	67 (82.7)	3.52 (1.58, 7.86)	3.56 (1.19, 10.69)*
	No	13 (5.6)	219 (94.4)	Ref.	Ref.
Being resuscitated	Yes	16 (14.5)	94 (85.5)	3.65 (1.64, 8.72)	3.69 (1.16, 11.75)*
	No	11 (5.4)	192 (94.6)	Ref.	Ref.
EBF	Not at all	4 (21.1)	15 (78.9)	4.69 (2.59, 14.47)	2.76 (0.69, 10.97)
	Initiated after 1 h	16 (9.8)	148 (90.2)	1.90 (0.71,6.21)	1.06 (0.29, 3.92)
	Initiated within 1 h	7 (5.4)	123 (94.6)	Ref.	Ref.

- *Ref. *Reference, *ANC *antenatal care, *SVD *spontaneous vaginal delivery, *IVD *instrumental vaginal delivery, *C/S *cesarean delivery, other complications = gestational diabetes mellitus, oligohydramnios and Rh incompatibility, *PROM *prolonged rapture of membrane, *EBF *exclusive breastfeeding, p value: *< 0.05, **<0.01

## Discussion

This study aimed to assess neonatal mortality and associated factors among neonates admitted to the neonatal intensive care unit (ICU) in the study setting. The magnitude of NM was 8.6%, with a 95% CI (5.8, 11.4), and complications during pregnancy, PROM, instrumental delivery, and resuscitation were significantly associated with NM. This study was similar to previous studies performed in Ethiopia, such as in Dilla (9.5%), GMH (7.7%), and Dire Dawa (11.4%) [25, 28, 29]. This study is also consistent with previous studies done in Iran (11.4%), Nigeria (10.5%), and Asmara Eritrea (6.5%) [11, 30, 31].

However, the magnitude of NM in this study was greater than that in other previous studies conducted in Pakistan (4.5%), India (5.0%) and Kenya (1.9%) [32–34]. This finding is also greater than studies reported in Addis Ababa, Ethiopia, which were_3.3% [24].

This difference might be due to variations in the types of data collection methods, study settings, and sample sizes used. Other reasons might be the differences in study design, study period and study subjects. In addition, the discrepancy could be due to variations in study settings. Unlike the current study settings (tertiary level), which had too many cases and many complicated referral situations, the majority of the studies mentioned above were conducted in low-level settings with limited neonatal flow; services may be more likely to be compromised when there are too many clients and complicated problems.

In contrast, the magnitude of NM in this study was lower than that reported in studies conducted in Nepal (17.6%, Tanzania (39.4%), Uganda (19.8%), and Sudan (21.9%) [35–38]. The finding of the present study is also lower than those of studies done in Woliso_17%, Wollega_15.3%, Amhara region_18.6%, and Jijiga_20.5% [14, 23, 39, 40]. This discrepancy might be attributed to differences in the study design, study settings, study subject differences, and socio demographic characteristics of the study participants. Previous studies in Tanzania and Uganda focused only on preterm neonates. However, in this study, the study population included all neonates who were admitted to the NICU during the study period.

Regarding factors associated with NM, the current study revealed that newborns whose mothers experienced complications during pregnancy were more likely to experience neonatal mortality than those whose mothers had no complications. This study is supported by studies done in America [41], the Republic of Haiti [42], and a systematic review and meta-analysis done in SSA [19]. This significant association is also consistent with studies done in Diredawa and Hiwot Fana Specialized University Hospital, Ethiopia [26, 29]. This association could be due to complications during pregnancy, such as PIH and APH, lead to fetal hypoxia and utero-placental dysfunction, which results in a nutritional and oxygen supply to the fetus, leading to preterm birth, IUGR, and LBW and then leads to neonatal death.

The current study revealed that neonatal mortality was greater in neonates born to PROM mothers than in their counterparts. This finding is consistent with a previous studies in Arba-Minch, Dire Dawa, and GMH [17, 29]. This might be due to PROM is associated with prematurity complications such as infection, risk of respiratory distress, and pulmonary hypoplasia, which may lead to NM.

The study also revealed that neonates who underwent resuscitation and that newborns delivered through instrumental delivery were more likely to die than their counterparts. Accordingly, newborns delivered through instrumental delivery were more than 5-fold and 3.7 times more likely to die than neonates who were delivered through spontaneous vaginal deliveries and newborns that were not resuscitated, respectively. This finding is supported by studies done in western Uganda [43], Somalia [13], Debiretabor and Assela Ethiopia [4, 44]. The use of instruments during birth might lead to soft tissue injuries and cranial hemorrhage, which could subsequently contribute to neonatal mortality [45]. Similarly, even resuscitation efforts aim to save lives; neonates who require resuscitation are often born with serious health conditions that necessitate immediate interventions. Thus, the presence of these severe problems and the challenges of effective resuscitation might contribute to the increased risk of death compared with neonates who do not require resuscitation.

### Limitation of the study

This study is limited by its retrospective nature and dependence on secondary data, which restricted the range of variables available. However, this approach allowed for the efficient use of existing information. In addition, the relatively small sample size may limit generalizability, but it enabled for a detailed and focused analysis.

## Conclusion

The present study finding revealed about 1 in 12 neonates admitted to the NICU did not survive. Complications during pregnancy, PROM, instrumental vaginal delivery, and neonates received resuscitation were significantly associated with neonatal mortality. As the so, stakeholders need to develop targeted interventions that address all identified risk factors into consideration.

### Recommendations

The Ministry of Health needs to strengthen initiatives to reduce NM by improving service utilization toward quality care and considering all risk factors. Healthcare providers working in the area should place priority on reducing NM among mothers with pregnancy-related complications and mothers with PROM by providing proper information about its risks and complications to their neonates. Even though instrumental vaginal deliveries are a key element of essential obstetric care, maternity care providers should be aware of key indications for instrumental delivery. Additional research using a different study design approach in different study settings is needed.

## Data Availability

The datasets generated and/or analyzed during the current study are not publicly available due to ethical restrictions of protecting study subjects’ privacy and confidentiality, but are available from the corresponding author on reasonable request.

## Abbreviations

AACAHB: Addis Ababa City Administration Health Bureau
AGMCH: Abebech Gobena Maternal and Children Hospital
GMH: Gandhi Memorial Hospital
MCSH: Menelik II Comprehensive Specialized Hospital
NM: Neonatal Mortality
TASH: Tikur Anbesa Specialized Hospital
